# Infectious Herpes Simplex Virus in the Brain Stem Is Correlated with Reactivation in the Trigeminal Ganglia

**DOI:** 10.1128/JVI.02209-18

**Published:** 2019-04-03

**Authors:** Jessica R. Doll, Richard L. Thompson, Nancy M. Sawtell

**Affiliations:** a University of Cincinnati, Department of Molecular Genetics, Biochemistry, and Microbiology, Cincinnati, Ohio, USA; b Cincinnati Children’s Hospital Medical Center, Division of Infectious Diseases, Cincinnati, Ohio, USA; University of California—Irvine

**Keywords:** hypercellular cuff, mouse ocular model, hyperthermic stress, in vivo reactivation, viral latency, virus recovery, sensory neuron

## Abstract

Herpes simplex virus (HSV) establishes latency in neurons of the peripheral and central nervous systems (CNS). Evidence is mounting that HSV latency and reactivation in the nervous system has the potential to promote neurodegenerative processes. Understanding how this occurs is an important human health goal. In the mouse model, *in vivo* viral reactivation in the peripheral nervous system, triggered by hyperthermic stress, has been well characterized with respect to frequency and cell type. However, characterization of *in vivo* reactivation in the CNS is extremely limited. Further, it remains unclear whether virus reactivated in the peripheral nervous system is transported to the CNS in an infectious form, how often this occurs, and what parameters underlie the efficiency and outcomes of this process. In this study, reactivation was quantified in the trigeminal ganglia (TG) and the brain stem from the same latently infected animal using direct assays of equivalent sensitivity. Reactivation was detected more frequently in the TG than in the brain stem and, in all but one case, the amount of virus recovered was greater in the TG than that detected in the brain stem. Viral protein positive neurons were observed in the TG, but a cellular source for reactivation in the brain stem was not identified, despite serially sectioning and examining the entire tissue (0/6 brain stems). These findings suggest that infectious virus detected in the brain stem is primarily the result of transport of reactivated virus from the TG into the brain stem.

**IMPORTANCE** Latent herpes simplex virus (HSV) DNA has been detected in the central nervous systems (CNS) of humans postmortem, and infection with HSV has been correlated with the development of neurodegenerative diseases. However, whether HSV can directly reactivate in the CNS and/or infectious virus can be transported to the CNS following reactivation in peripheral ganglia has been unclear. In this study, infectious virus was recovered from both the trigeminal ganglia and the brain stem of latently infected mice following a reactivation stimulus, but a higher frequency of reactivation and increased titers of infectious virus were recovered from the trigeminal ganglia. Viral proteins were detected in neurons of the trigeminal ganglia, but a cellular source of infectious virus could not be identified in the brain stem. These results suggest that infectious virus is transported from the ganglia to the CNS following reactivation but do not exclude the potential for direct reactivation in the CNS.

## Abstract (precis)

Latent herpes simplex virus (HSV) DNA has been detected in the central nervous systems (CNS) of humans postmortem, and infection with HSV has been correlated with the development of neurodegenerative diseases. However, whether HSV can directly reactivate in the CNS and/or infectious virus can be transported to the CNS following reactivation in peripheral ganglia has been unclear. In this study, infectious virus was recovered from both the trigeminal ganglia and the brain stem of latently infected mice following a reactivation stimulus, but a higher frequency of reactivation and increased titers of infectious virus were recovered from the trigeminal ganglia. Viral proteins were detected in neurons of the trigeminal ganglia, but a cellular source of infectious virus could not be identified in the brain stem. These results suggest that infectious virus is transported from the ganglia to the CNS following reactivation but do not exclude the potential for direct reactivation in the CNS.

## INTRODUCTION

Herpes simplex virus (HSV) infection occurs at the body surface and results in establishment of a latent viral reservoir in neurons of both the peripheral and central nervous systems (1). The ability of the virus to persist in a latent state has contributed to widespread infection in the human population (2). Periodic reactivation allows for spread to new hosts but has also been attributed to neurodegenerative diseases, such as Alzheimer’s disease (3, 4), epilepsy (5), and multiple sclerosis (6). That reactivation occurs in peripheral ganglia has been well established, but the ability of latent genomes in the central nervous system (CNS) to reactivate and the long-term consequences of host/viral responses in the CNS following a reactivation stimulus are not well understood.

Detection of HSV DNA in human CNS tissue postmortem has been variable, ranging from 2 to 100% of samples tested (1, 7–10). This extreme variability could be due to differences in cohort size, amount of tissue sampled, and/or the way samples were collected and analyzed between the multiple studies. The presence of HSV genomes in the CNS raises the possibility that reactivation can occur directly in the CNS, but it does not exclude the possibility that reactivated virus can be transported from the trigeminal ganglia (TG) to the CNS. TG neurons are pseudounipolar with processes projected to the body surface, as well as the trigeminal nucleus located in the brain stem. From the trigeminal nucleus, neuronal projections reach the thalamus and then the sensory cortex, providing a route for HSV to move throughout the brain and potentially play a role in CNS disorders (reviewed in reference 3). Determining the ability of HSV genomes to reactivate directly in the CNS and/or the propensity for reactivation in the periphery to reach this site is an important step for determining a mechanism whereby long term HSV infection could contribute to neurodegenerative disease and to develop strategies to prevent neurological damage.

The mouse model represents a tractable system for investigating the impact of HSV reactivation on the CNS *in vivo*. Previous studies have rarely recovered infectious virus in CNS tissues by traditional explant assays for reactivation competency (11–15), which has led to the conclusion that HSV reactivation is very inefficient or does not occur in the CNS. However, adult CNS tissues have limited viability *ex vivo*; deterioration of neurons in brain slice cultures has been reported as early as 4 h postexplantation, and while individual neurons can be isolated from neonatal CNS and cultured for extended time periods, the estimated survival of neurons in adult CNS tissues *ex vivo* is approximately 6 to 12 h (16–19). The limited viability of CNS tissues in explant could be an underlying reason for the limited recovery of infectious virus from latently infected, explanted CNS tissue (12, 20–22).

An inaugural study used C57BL/6 mice to compare *in vivo* reactivation in the brain stem to reactivation in the TG (23). Detection of a low frequency of reactivation in the TG relative to the brain stem led the authors to conclude that reactivation occurred in the brain stem independently of the TG. Interestingly, it has been proposed that the lesions and tangles characteristic of Alzheimer’s disease may originate from neurons located in the brain stem (24–26). Amyloid precursor protein, the parent molecule of amyloid beta that is a major component of Alzheimer’s plaques, has altered subcellular localization in HSV-infected cells and has also been found to directly interact with HSV particles (27).

Although the conclusions of the previous study support the hypothesis that HSV reactivation can occur in the CNS, the assay used for virus detection was not quantitative and relied on a two-step culturing method and a cellular source for infectious virus production was not identified (23). The low frequency of infectious virus detected in the TG was inconsistent with the high frequency of reactivation we have previously measured in the TG following a reactivation stimulus in Swiss-Webster mice (28–30), which prompted us to reexamine reactivation in the trigeminal ganglia directly compared to reactivation in the brain stem using C57BL/6 mice.

## RESULTS

### Infection and establishment of latency.

**FIG 1:**
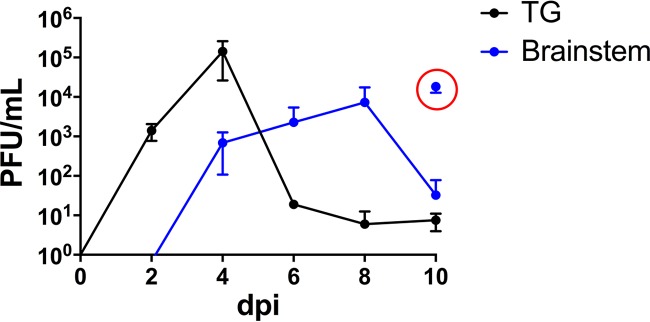
TG and brain stem infection of C57BL/6 mice with HSV-1 McKrae. C57BL/6 female mice were infected with HSV-1 McKrae as described in Materials and Methods. Tissues from at least three mice were harvested at the indicated times during acute infection. TG (black) and brain stem (blue) samples were homogenized (whole) to determine infectious virus titers. The red circle indicates samples obtained from mice (*n* = 3) displaying signs of encephalitis (e.g., hunched posture) on day 10 p.i.

C57BL/6 female mice were infected with HSV-1 strain McKrae, as described in Materials and Methods, and viral replication efficiency and the amount of latency established in the TG and brain stem were determined from a minimum of three mice at each time point. Infectious virus titers peaked in the TG on day 4 postinfection (p.i.), achieving titers of ∼10^5^ PFU/ganglia and declining over the next 6 days. In the brain stem, titers were ∼10^3^ PFU/brain stem on day 4 p.i. and continued to rise until day 8 p.i., peaking at ∼10^4^ PFU (Fig. 1). Titers were variable during the end of acute infection; high levels of infectious virus were recovered from mice displaying signs of encephalitis (e.g., hunched posture) that likely would have succumbed to infection (mortality rate, 45% [35/78]), while only low levels of infectious virus were recovered from mice not displaying such signs (Fig. 1). At >45 days p.i. (dpi), latent genomes were quantified in the TG and brain stems from three mice by quantitative reverse transcription-PCR, as previously described (31). There were 512 ± 46 latent genomes/50 ng of DNA recovered in the TG compared to 115 ± 49 latent genomes/50 ng of DNA detected in the brain stem (Student's *t* test; *P* = 0.0012).

### Recovery of infectious virus is related to tissue weight.

In order to directly compare the frequency of reactivation and the amount of infectious virus produced in the brain stem and TG post-hyperthermic stress (phs), a quantitative assay with equivalent sensitivity to that utilized to quantify virus reactivation in the TG was required to quantify virus reactivation in the brain stem. We anticipated that as in the TG, low levels of virus would be generated from a very limited number of cells in the brain stem. Alternatively, reactivated virus transported from TG into the brain stem would be anticipated to be at or below the levels detected in the TG. One obvious difference between TG and brain stem is the size of the tissue, the weight of the brain stem is ∼6 times that of a TG pair, 0.081 ± 0.021 g versus 0.015 ± 0.005 g, respectively. This difference in tissue size would be expected to dramatically limit recovery of infectious virus and also detection of any rare viral protein positive cells in the brain stem.

To test the effect of tissue weight on virus recovery, brain stems were harvested from uninfected mice and divided into halves, fourths, or maintained as whole pieces. Each sample size was weighed and samples were spiked with approximately 150, 75, or 20 PFU, representative of the low levels of virus typically associated with a reactivation event *in vivo* (28). Spiked tissues were homogenized in 1 ml of media, centrifuged to pellet cell debris, and 500 μl of the supernatant were plated in each of two wells of a six-well plate, as detailed in Materials and Methods. After absorption, wells were overlaid with 1% carboxymethyl cellulose. Two days after plating, rinsed wells were stained with crystal violet and plaques were counted. Recovery of infectious virus in the context of the tissue homogenate was determined by comparing the number of plaques detected in spiked samples to that detected in the inoculum plated in media alone. Recovery was proportional to tissue weight; >25% recovery of input virus was observed in 44% (8/18) spiked samples weighing <0.025 g, while only 6.6% (1/15) of samples weighing >0.025 g reached this efficiency. Importantly, false negatives were observed at a rate of 0% (0/18) in samples <0.025 g compared to 20% (3/15) in samples >0.025 g. The percent recovery was significantly different between the lowest (20 PFU) and the highest PFU (150 PFU) tested, *P* = 0.009, one-way analysis of variance (ANOVA).

These data revealed that tissue weight could influence infectious virus recovery in the range of viral titers observed during *in vivo* reactivation in the TG. To determine the relative percent recovery of infectious virus in the brain stem compared to the TG when tissue weights were similar, brain stems were harvested from 13 mice and sagitally sectioned into six pieces (weights are shown in Table 1). Each piece was homogenized in 1 ml and spiked by adding 50 μl of a stock containing approximately 150, 75, or 20 PFU/50 μl (confirmed titers, 154 ± 11, 77 ± 6, and 21 ± 10 PFU/50 μl) of 17VP16pLZ (30). This *lacZ* marker bearing virus was utilized so that exogenous virus could be distinguished from endogenous virus in spiking experiments done in the context of latent infection pre- and post-hyperthermic stress. As shown in Table 1, plaques were detected in every sample. The mean recovery observed in each of three groups was not different at the same input PFU and not different from the mean recovery in the spiked homogenized TG (*P* = 0.99, one-way ANOVA).

**TABLE 1:** Recovery of infectious virus^*a*^

Parameter	BS, 20 PFU	BS, 20 PFU	BS, 20 PFU	TG, 20 PFU	BS, 75 PFU	TG, 75 PFU	BS, 150 PFU	BS, 150 PFU	BS, 150 PFU	TG, 150 PFU
Input PFU	21	21	21	21	77	77	154	154	154	154
Recovered PFU	8	2	6	8	9	29	70	57	43	97
	4	11	7	5	12	21	83	70	57	78
	6	4	4	10	21	18	83	90	50	80
	6	5	5	4	17	27	33	93	90	31
	4	15	6	6	33		50	40	80	52
	1	4	6		24		47	43	73	
	8	7			22		67	103		
	4	4			26		50	80		
	3	4			23		67	30		
	8	5			26		137	77		
	11	7			21		87	63		
	7	3			27		47	57		
Mean tissue wt (g) ± SD	0.0114 ± 0.0020	0.0107 ± 0.0022	0.0139 ± 0.0048	0.0155 ± 0.0047	0.0119 ± 0.0046	0.0137 ± 0.0035	0.0119 ± 0.0013	0.0113 ± 0.0014	0.0103 ± 0.0007	0.0167 ± 0.0078
Mean % recovery ± SD	27.78 ± 13.13	28.17 ± 17.64	26.98 ± 4.92	31.43 ± 11.47	28.24 ± 8.56	30.85 ± 6.65	44.42 ± 17.87	43.45 ± 14.74	42.53 ± 11.92	43.89 ± 16.89

a Uninfected brain stems were divided into six parts and individually weighed. TG pairs were also harvested and weighed. Each brain stem (BS) or TG sample was spiked with approximately 20, 75, or 150 PFU of 17VP16pLZ and homogenized as described in Materials and Methods. The input PFU were also directly plated alongside tissue samples onto RSC. Each BS column represents an independent experiment. Recovery in paired TG was pooled from 2 to 3 experiments. Plates were stained with crystal violet 2 to 3 days later, and the numbers of PFU recovered were counted. The number of PFU recovered in each sample was divided by the average PFU detected by directly plating the input virus, and the results are presented as the mean percent recovery ± the standard deviation for each experiment.

To test the possibility that the latently infected and/or post-hyperthermic stress tissue environment would alter recovery, brain stems from five latently infected mice, pre- and post-hyperthermic stress, were spiked with approximately 20 PFU (confirmed titer 21 ± 10 PFU/50 μL) of 17VP16pLZ (30). Recovery of 17VP16pLZ in brain stems pre-hyperthermic stress was on average 34% (range 2 to 16 PFU) and at 24 h post-hyperthermic stress was on average 28% (range 1 to 19 PFU). Infection and hyperthermic stress did not appear to significantly alter the level of recovery compared to uninfected tissue.

### In vivo reactivation can be detected in the brain stem.

The direct approach described above was used to evaluate reactivation in the brain stem post-hyperthermic stress of C57BL/6 female mice latently infected with HSV McKrae (>45 days pi). In two independent experiments (*n* = 5, *n* = 6), TG and brain stems were harvested at 20 to 24 h phs and directly processed for the detection of infectious virus. Brain stems were divided into six pieces, and each piece was individually homogenized, as described above and in Materials and Methods. Infectious virus was detected in 82% (9/11) of the TG and 45% (5/11) of the brain stems at 20 to 24 h phs (*P* = 0.037, Student's *t* test) (Table 2). Infectious virus was not detected in brain stems from mice in which the TG were infectious virus negative, although this included only two TG. With the exception of one mouse, more infectious virus was recovered from the TG than the brain stem (Table 2). Infectious virus was not detected in the TG or brain stem prior to hyperthermic stress (0/5 mice).

**TABLE 2:** Reactivation in the brain stems of C57BL/6 mice^*a*^

Expt and TG pair	PFU						
	BS total	BS-1	BS-2	BS-3	BS-4	BS-5	BS-6
Expt 1							
149	2	2	0	0	0	0	0
87	12	3	9	0	0	0	0
1	0	0	0	0	0	0	0
0	0	0	0	0	0	0	0
105	8	1	7	0	0	0	0
							
Expt 2							
0	0	0	0	0	0	0	0
26	0	0	0	0	0	0	0
4	0	0	0	0	0	0	0
63	0	0	0	0	0	0	0
15	70	14	3	50	3	0	0
51	4	1	1	2	0	0	0

a C57BL/6 mice latently infected with McKrae were induced to reactivate by hyperthermic stress. The results are presented from two independent experiments. Tissues were harvested at 20 to 24 h phs and processed for the detection of infectious virus by the direct, quantitative approach. Shown are the PFU recovered in the TG and brain stem (BS; processed in six pieces) from each mouse. The BS total is the total PFU detected in the BS-1 through BS-6 sections for each mouse.

### The two-step assay has a high risk for contamination.

It has previously been reported that recovery of infectious virus in brain stem tissue can be enhanced by culturing homogenized samples in a 3-ml volume for 3 days, harvesting wells, and replating the samples on new monolayers for an additional 3 days (23). Overlay (used to neutralize released virus and allow for quantification of plaque forming units) was limited to the second culture step only. The authors reported that when brain stem samples were spiked with 10 PFU, cytopathic effect (CPE) was not detectable during the first culture step but was observed following replating on fresh monolayers (23). Due to the reported enhanced sensitivity of this approach, we compared the two-step assay to our direct method for detection of HSV reactivation in the brain stem.

**FIG 2:**
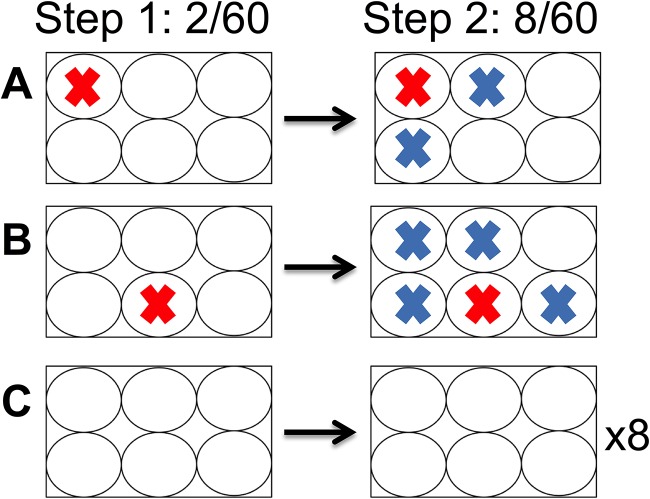
Probable contamination resulting from two-step culture assay. Mice latently infected with McKrae were subjected to hyperthermic stress to induce HSV reactivation. At 24 h phs, brain stems were harvested and processed by the two-step culture assay as described in Materials and Methods. During the first 3 days of plating, a viral CPE was observed in 2/60 samples (red “X”). All wells were harvested and replated. After 3 additional days of incubation, an additional six positive samples were detected (blue “X”). (A and B) Locations of positive wells during the first plating and spatial relation of additional positive wells after replating. (C) Eight of the six-well plates did not contain positive samples at any time during the two-step assay.

Five C57BL/6 female mice latently infected with HSV McKrae were induced to reactivate using hyperthermic stress (29). TG and brain stems were harvested at 20 to 24 h phs. Brain stems were divided into six parts, and TG and brain stem pieces were directly homogenized. Homogenates were plated onto RSC monolayers in six-well plates and maintained in culture according to the two-step detection assay and as described in Materials and Methods. During step 1, 2/60 wells (one well on each of two plates) had evidence of CPE. Consistent with the previous report (23), the second step of this assay resulted in additional positive wells, 8/60 or a 4-fold enhancement. However, wells that became positive during the second step were always spatially related to the wells that were positive in the first step (Fig. 2). Strikingly, none of the 48 wells on the eight plates which contained no CPE during step 1 developed CPE during step 2. The correlation between a positive well in step 1 and additional positive wells in step 2 on a given plate was significant (*r* = 0.9631; Pearson’s correlation, *P* < 0.0001). This raised the possibility that the second step of the assay was a result of cross-contamination and not a delayed ability to detect virus generated during the *in vivo* reactivation.

To test the potential for contamination using the two-step assay, virus expressing fluorescent marker during acute infection was utilized. This allowed us to visualize and count plaque development and spread while the assay was ongoing. Two six-well plates were spiked with ∼9 PFU of OK12 fluorescent virus (32); one plate with two positive wells and the second plate with one positive well, and monitored daily. At 48 h p.i., small green fluorescent protein (GFP)-positive viral plaques were observed in the three spiked wells (5, 4, and 6 PFU), which spread throughout each well by 72 h p.i. No signs of infection (neither CPE nor GFP positivity) were detected in the nine negative-control wells. At this time (72 h p.i.), both infected and uninfected wells were individually harvested and the 3 ml/well volume replated onto new monolayers. After 24 h of incubation, the sample was removed, and each well was overlaid. The replated samples harvested from one of the spiked plates now had two additional positive wells, which presumably became contaminated in the first 24 h of the second incubation period (38 and >100 PFU detected 3 days postreplating) from aerosolized virus originating from positive spiked wells. The plate with one positive well did not develop additional positive wells, indicating that contamination may occur sporadically. To further test this possibility, the basic experiment was repeated with similar outcomes multiple times by independent investigators to ensure that this was not simply based on poor technical skill. In an additional experiment, individual 60-mm dishes instead of six-well plates were utilized. In this case, as observed with replating wells from six-well plates with no CPE during step 1, no additional positive samples were observed after the second plating. We conclude that in our hands, the absence of overlay in this two-step method presents a high risk for contamination of negative samples on a six-well plate with infectious virus.

Due to the risk for contamination when culturing high titers of PFU in a large volume near negative wells, samples with evidence of CPE during the first culture step were not replated. Across three additional reactivation experiments, 140 samples that appeared reactivation negative during the first culture step were replated. Importantly, infectious virus was not detected in any of these samples (0/140). This demonstrates that if infectious virus is present in the brain stem, it can be detected by 3 days postplating (similarly to the TG), and extensive culturing time does not result in additional reactivation positive samples.

### The source of infectious virus in the brain stem is undetermined.

**FIG 3:**
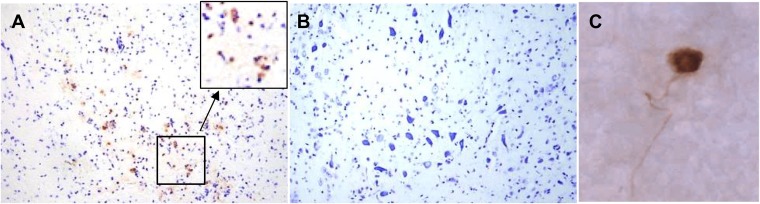
Viral proteins were not detected in the brain stem post-hyperthermic stress. C57BL/6 female mice were infected with McKrae. (A) At 8 dpi, mice displaying symptoms of encephalitis were sacrificed, and the brain stems were processed for the detection of viral proteins (brown) and counterstained with cresyl violet. The box shows the area at a higher magnification. (B and C) Latently infected mice were induced to reactivate by hyperthermic stress. At 24 h phs, the brain stems and TG from six mice were harvested and processed for the detection of viral proteins. Viral proteins were not detected in the brain stem (B), but positive neurons (brown) were identified in the TG by whole-ganglion immunohistochemistry (WGIHC) (C).

Infectious virus in the brain stem could be the result of direct reactivation from latently infected neurons in the brain stem, could result from transport of infectious virus from the TG to the brain stem, or both. The detection of rare reactivating neurons in the TG has been greatly enhanced by a method for immunohistochemical detection of viral proteins in the whole ganglia (28). However, this protocol cannot be directly applied to the brain stem because of the limits of penetration of the antibody reagents into tissue. Therefore, immunohistochemical analysis of sectioned brain stems was utilized to detect viral protein expression in brain stems at 24 h phs from mice latently infected with HSV-1 McKrae. Six brain stems were fixed overnight in 4% formaldehyde, divided in half sagitally, and subsequently embedded and sectioned at 10 μm. Serial sections were collected on slides and sections on each slide were assayed for viral proteins as detailed in Materials and Methods. Sections of brains from encephalitic mice that were infected with McKrae and harvested at 8 dpi were also processed as positive controls, and sections of uninfected brain stems were used as negative controls. The embedding and sampling strategy are detailed in Materials and Methods. As an initial approach, every fourth slide (120 μm/brain stem) was analyzed for HSV protein-expressing cells. No viral protein-positive cells were detected. Additional slides were tested for HSV protein expression until each entire brain stem (approximately 3.2 mm per brain stem) had been examined. Viral protein-positive cells were not detected in any of the brain stems at 24 h phs, in contrast to the encephalitic brains, which contained viral protein-positive neurons on nearly every section (Fig. 3). The TG were also tested for HSV protein expression, and a total of 24 viral protein-positive neurons were detected in 12 ganglia (8/12 [75%] ganglia positive).

## DISCUSSION

The presence of even low levels of infectious HSV in the CNS has been proposed to have significant implications for human health (reviewed in reference 3). Periodic reactivation events have the potential to result in neuronal loss and inflammation (29, 33, 34), which could contribute to neurodegenerative diseases (4, 35–37). The long-term impact of HSV infection is not well understood, and whether or not HSV reactivation occurs directly in the CNS has been unclear (reviewed in references 3 and 38). Investigating reactivation in the brain stem is a reasonable first step to understanding HSV pathology in the CNS, since it has been proposed that lesions in the front of the brain, characteristic of Alzheimer’s disease, may originate from neurons located in the brain stem (24, 25).

Standard plaque assays have rarely recovered infectious HSV in CNS tissue postreactivation stimulus *in vivo* (12, 14, 15, 20–22), which may be due to the large size of tissue processed. In this study, we have expanded on previous work that detected reactivation in the brain stem (23) by developing a quantitative assay for infectious virus detection that allowed direct comparison to reactivation in the TG. We have shown that the two-step cultivation assay used previously for detecting reactivation in the brain stem (23) has a high risk for contamination in our hands, even with extreme care. Using a direct assay to detect and quantify reactivation, we found that infectious virus can be detected in the brain stem post-hyperthermic stress, but virus reactivation was detected at a higher frequency and at increased titers in the TG.

Localization of viral protein positive neurons would be necessary to definitively conclude that reactivation can occur directly in the brain stem. Given that reactivation was detected in 45% of brain stems at 20 to 24 h phs with McKrae in the present study, it would be predicted that two or three of six brain stems would contain at least one viral protein positive neuron (or other cell type) at 24 h phs if reactivation was occurring directly in the CNS. The entire brain stems from six mice latently infected with McKrae and harvested at 20 to 24 h phs were serially sectioned and processed for HSV protein expression. However, in these 160 sections, which included the full 3.2-mm thickness of each brain stem, viral protein-positive cells were not detected. Due to the large size of the brain stem, inherent tissue loss during processing, and the predicted rarity of a reactivation event, this outcome does not exclude reactivation occurring directly in the brain stem, but it does offer support for the hypothesis that infectious virus is being transported from a reactivation event in the TG.

Although a cellular source for reactivation in the brain stem was not identified, the detection of infectious virus in the CNS postreactivation stimulus confirms an earlier report (23), strengthening the potential role of long-term HSV infection as an additional risk for the development of neurodegeneration (reviewed in reference 4). Interestingly, if reactivation occurs primarily in the periphery, prophylactic treatment strategies with antiviral drugs such as acyclovir have significant therapeutic potential toward preventing damage in the CNS (reviewed in references 4 and 39) by inhibiting production of infectious virus in the periphery and therefore preventing transport to the CNS. Further investigation is necessary to understand the long-term consequences of periodic reactivation events in the nervous system.

## MATERIALS AND METHODS

### Cells and viruses.

Rabbit skin cells (RSC; originally obtained from B. Roizman at the University of Chicago) were maintained in minimal essential medium supplemented with 5% newborn calf serum and incubated at 37°C in a 5% CO_2_ incubator. Virus stocks of HSV-1 strain McKrae (originally obtained from S. Wechsler at Mount Cedar Sinai Medical Center Research Institute), HSV-1 strain 17syn+ (originally obtained from C. Preston at the MRC Virology Unit in Glasgow, Scotland), HSV-1 strain OK12 (32), and 17VP16pLZ (30) were generated by routine propagation on RSC monolayers. Infected RSC were harvested, frozen, and thawed three times, and the titer was determined by serial 10-fold dilution plaque assay on RSC monolayers. After a 2-h incubation period, infected monolayers were overlaid with medium containing 1% carboxymethyl cellulose and stained with crystal violet 2 to 3 days later. Stocks were aliquoted and stored at –80°C.

### Inoculation of mice.

All procedures involving animals were approved by the Children’s Hospital Institutional Animal Care and Use Committee and were in compliance with NIH guidelines. Animals were housed in American Association for Laboratory Animal Care-approved quarters. C57BL/6, male or female as indicated (bred in-house; 6 to 8 weeks old), were anesthetized by intraperitoneal injection of sodium pentobarbital (50 mg/kg of body weight) prior to inoculation. A 10-μl drop containing 1 × 10^5^ PFU of McKrae was placed onto each scarified corneal surface (40). TG and brain stems were homogenized as whole tissues, and samples were serially diluted and plated on RSC to determine titers from a minimum of three mice at each time point (2, 4, 6, 8, and 10 dpi) during acute infection. Mice demonstrating signs of CNS distress (e.g., hunched posture) were euthanized, and tissues were collected.

### Quantification of viral genomes by real-time PCR.

Isolation and quantification of total viral genomes by real-time PCR was performed as detailed previously with some modifications (31). The trigeminal ganglia and brain stems from three mice maintained for >45 days postinfection were individually processed to determine latent viral genomes. Five microliters containing 50 ng of sample DNA was combined with 15 μl of QuantiTest SYBR Green PCR mix (Qiagen) containing 10 pmol of TK primers and assessed under the conditions previously described (31). A Roche 480 II LightCycler system was used to measure DNA amplification in real time.

### *In vivo* reactivation.

Latent HSV was induced to reactivate *in vivo* by the hyperthermic stress method (29, 40, 41). In brief, each mouse was placed in a restrainer and suspended in a 42.5 to 42.8°C water bath for 10 min. Mice were subsequently towel dried and placed in a 35°C incubator for 20 to 30 min to prevent hypothermia. This procedure was performed 3 consecutive times spaced 2.5 h apart. At the indicated times after the initial stress, the TG were removed and processed for the detection of infectious virus or immunohistochemical analysis.

### Detection of reactivated virus (quantitative).

At the indicated times post-hyperthermic stress, TG pairs were removed and homogenized as described previously (40). Brain stems were dissected and sagitally divided into six sections. Each section was individually homogenized in 1 ml using one homogenizer per brain stem (six parts). All samples were briefly centrifuged to remove cellular debris. The supernatant was plated onto two wells of a six-well plate seeded the day before with RSC. It was observed that dividing the supernatant over two wells could increase recovery as much as 2-fold compared to plating in a single well. Plates were incubated for 3 h to allow for viral absorption and then rinsed with fresh media. The next morning, RSC monolayers were overlaid with medium containing 1% carboxymethyl cellulose and stained with crystal violet approximately 2 to 3 days later.

### Two-step assay for the detection of reactivated virus.

TG pairs and brain stems were harvested and brain stems were sagitally divided into six pieces. Each TG pair and brain stem piece was homogenized in 3 ml of medium. Homogenates were briefly centrifuged, and the supernatant was plated on one well of a six-well plate. Samples were cultured for 3 days at 37°C in a 5% CO_2_ incubator and monitored for CPE. Each well was then harvested and plated on a new well seeded with RSC the previous day. After 24 h, the medium was removed and replaced with overlay (medium containing 1% carboxymethyl cellulose). At 72 h postreplating, the plates were stained with crystal violet.

### Antibodies and immunohistochemistry.

HSV proteins were detected in whole ganglia as described previously (28). Primary antibody used was rabbit anti-HSV (AXL237; Accurate) diluted 1:3,000 and secondary antibody used was horseradish peroxidase-labeled goat anti-rabbit (Vector) diluted 1:500. Color development was achieved by exposing ganglia to a 0.1 M Tris (pH 8.2) solution containing 250 μg of diaminobenzidine (Aldrich)/ml and 0.004% H_2_O_2_ for approximately 5 min. Tissues were cleared in glycerol to aid in visualization of the HSV protein-positive neurons.

Encephalitic brain stems harvested at 8 dpi, or brain stems harvested at 24 h phs from latently infected mice, were directly placed in 4% formaldehyde overnight. Brain stems were then dehydrated in a graded ethanol series, cleared in xylene, and paraffin embedded. Blocks were serially sectioned at 10 μm, and consecutive sections were placed on Superfrost Plus slides (Fisher Scientific). Slides were deparaffinized, rehydrated, and examined for HSV protein expression using the antibodies described above. Color development was achieved by exposing slides to the solution of diaminobenzidine (Aldrich). After viewing slides for positive reaction product, all slides were subsequently counterstained with cresyl violet (Sigma). All slides were viewed under an Olympus BX40 microscope and photographed with AxioCamHRc (Zeiss).

### Histochemical detection of β-galactosidase activity.

When HSV-1 strain 17VP16pLZ was used for determining infectious virus recovery in spiked samples, RSC plates were rinsed in phosphate-buffered saline (PBS) pH 7.2 and fixed in 4% formaldehyde for 5 min. The plates were then rinsed with PBS and exposed to a solution containing 5 mM potassium ferricyanide, 5 mM ferrocyanide, 2 mM magnesium chloride, and 0.1 μg of X-Gal (5-bromo-4-chloro-3-indolyl-β-d-galactopyranoside) buffer per ml of PBS for 3 h.

### Statistical analysis.

Statistical analyses were performed using Prism software (GraphPad Software, San Diego, CA). A *P* value of <0.05 was considered significant.
